# A technique for total skin electron therapy (TSET) of an anesthetized pediatric patient

**DOI:** 10.1002/acm2.12457

**Published:** 2018-09-29

**Authors:** Tomas Kron, Grace Donahoo, Peta Lonski, Greg Wheeler

**Affiliations:** ^1^ Peter MacCallum Cancer Centre Melbourne Australia; ^2^ Sir Peter MacCallum Cancer Department Melbourne University Melbourne Australia

**Keywords:** cutaneous lymphoma, *in vivo* dosimetry, pediatric oncology

## Abstract

**Purpose:**

Total skin electron therapy (TSET) is a technique to treat cutaneous lymphomas. While TSET is rarely required in pediatric patients, it poses particular problems for the delivery. It was the aim of the present work to develop a method to deliver TSET to young children requiring anesthetics during treatment.

**Methods:**

A customized cradle with a thin window base and Poly(methyl‐methacrylate) (PMMA) frame was built and the patient was treated in supine position. Two times six fields of 6 MeV electrons spaced by 60° gantry angles were used without electron applicator and a field size of 36 × 36 cm^2^. The two sets of six fields were matched at approximately 65% surface dose by rotating the patient around an axis 30 cm distance from beam central axis, effectively displacing the two sets of fields in sup/inf direction by 60 cm. Electron energy was degraded using a 12 mm PMMA block on the gantry. Focus to skin distance was maximized by displacing the patient in opposite direction of the beam resulting in a different couch position for each field.

**Results:**

A 2‐yr‐old patient was treated in 12 fractions of 1.5 Gy over 2.4 weeks. Dose to skin was verified daily using thermoluminescence dosimetry and/or radiochromic film. The treatment parameters were adjusted slightly based on *in vivo* dosimetry resulting in a dose distribution for most of the treatment volume within ±20% of the prescribed dose. Six areas were boosted using conventional electron therapy.

**Conclusion:**

TSET can be delivered to pediatric patients using a customized couch top on a conventional linear accelerator.

## 1 INTRODUCTION

Total skin electron therapy (TSET) is a radiation technique used for more than 50 yr to treat cutaneous T‐cell lymphomas (CTCL).^1^ These lymphomas are relatively rare^2^ but can have devastating effects on affected individuals. Mycosis fungoides is a type of CTCL often associated with a red rash of large parts of the patients’ skin. One effective treatment option is the use of electron radiation to treat the surface of the patient.

Irradiation of large parts or all of a patient's skin is technologically challenging and several techniques have been developed over the years.^3^, ^4^ However, modern techniques such as intensity modulated radiation therapy (IMRT) and image guidance had very little impact on the development of TSET techniques and the report of the American Association of Physicists in Medicine (AAPM) task group 30 of 1988 is still widely used as a guidance.^5^ Most treatment techniques rely on extended source to skin distance (SSD) to cover larger areas of the patient. At a distance of about 2 m from isocentre, it is possible to cover the whole length of a patient standing up using two angled radiation beams (up and down matched at about 65% dose). To cover the whole circumference of the patient, the patient is rotated continuously^4^, ^6^ or in typically six increments (“Stanford” technique).^7^

Electron energy used varies between 4 and 10 MeV in the literature — once oblique incidence and distance are taken into account this results in the desired depth of usually between 5 and 10 mm receiving 80% of the radiation dose. It is also accepted that not all parts of the body will equally be irradiated and *in vivo* dosimetry measurements are commonly employed to assess which parts of the anatomy need boosting and by what dose.^8^

Mycosis fungoides occurs mainly in adults above the age of 20. As such reports on techniques used for TSET in children are rare and mostly confined to case reports as a recent review by S. Malgorzata shows.^9^ The review identified seven reported cases in the literature based on several reports.^10^, ^11^, ^12^, ^13^, ^14^ Techniques used included treatment on the floor and the design of a specialized frame holding the child in an upright position at a distance.^12^

We are reporting a different technique that utilizes lateral and vertical couch movement to maximize the SSD for the beam in each direction by moving the couch position away from the radiation source. Combined with a custom‐made thin tabletop this allows the patient to be treated in supine position for the whole of the treatment which was considered essential for safe anesthetics.

## 2 MATERIALS AND METHODS

### 2.A Patient and prescription

The patient was a 2‐yr‐old female to be treated with total skin electron irradiation. The patient was approximately 90 cm tall with average body dimensions of 10 × 20 cm (ant/post × left/right). The treatment was to be delivered under general anesthetics and the medical team strongly suggested supine positioning with minimal patient movement between beams. Treatment was to be performed on a Varian Trilogy linear accelerator (Varian Medical Systems, Palo Alto, CA, USA).

The prescription was 18 Gy to the skin surface in 12 fractions given five times per week (1.5 Gy per fx). Dose variations on skin should be minimized with dose per fraction variations between 1.1 and 1.8 Gy deemed the maximum range acceptable by the clinician. Areas of low dose were to be identified using *in vivo* dosimetry and boosted using conventional electron irradiation.

### 2.B Technique

**Figure 1:**
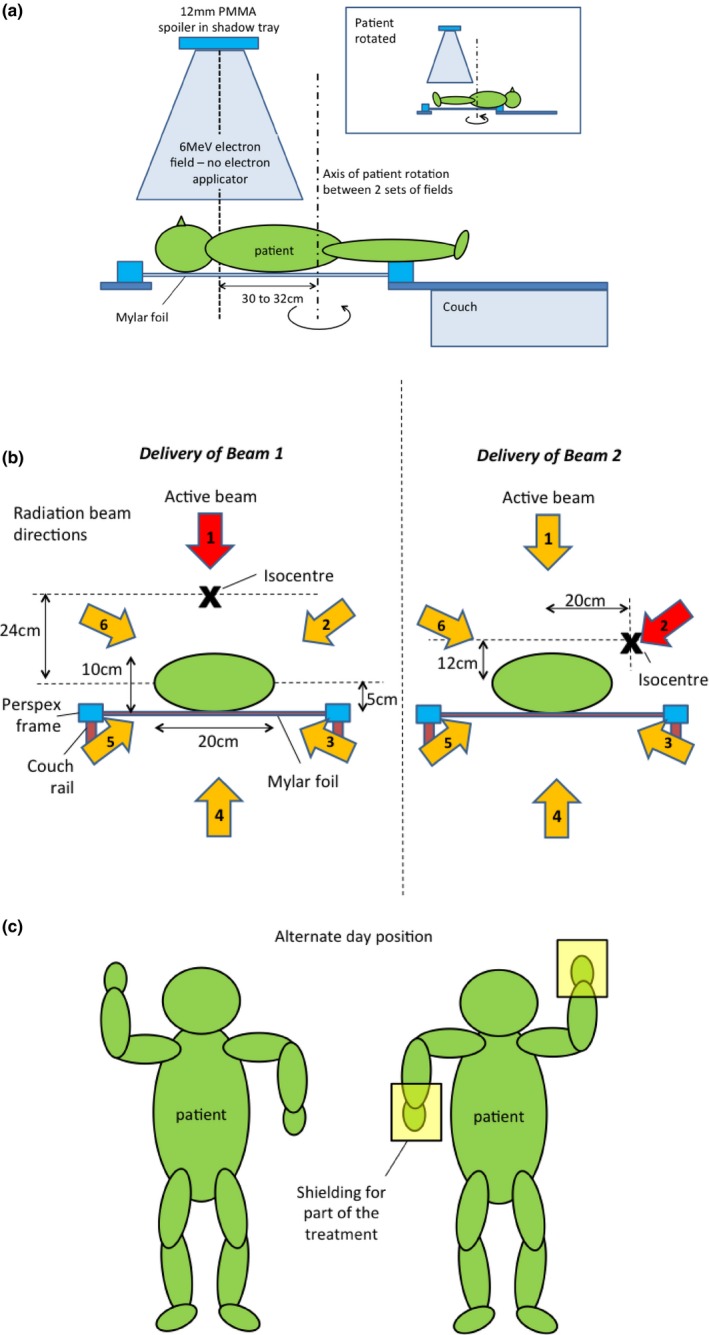
Patient setup for TSET. (a) Side view: the insert shows the patient rotated for the second set of 6 electron fields; (b) view from the feet: The isocentre of the linac is shown for two of the six couch positions for two of the six electron fields in each of the patient's position. The couch shifts were chosen so the centre of the patient assumed to be 5 cm above the couch top is always at a distance of 124 cm to the radiation source. For the posterior beams, the couch rails were adjusted to minimize their impact on dose; (c) anterior view: the position of PMMA hand shielding which was applied for parts of the treatment to improve dose homogeneity is indicated. Eyes were shielded for the whole treatment; toenail shielding was used from the third fraction onwards while fingernail shielding was added after fraction 8. Except for the hands, all shielding was manufactured in house and made from lead.

**Figure 2:**
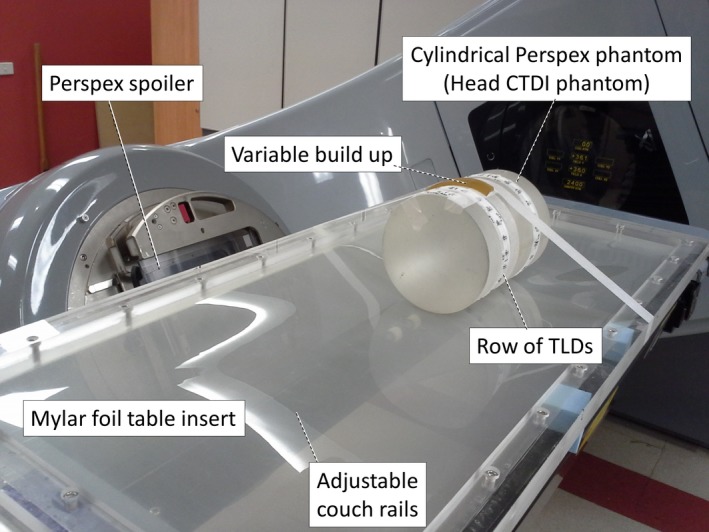
Photograph of TLD measurements for commissioning of the treatment technique. Shown is a 16 cm diameter PMMA phantom with a “ring” of TLDs on the customized thin window tabletop. The adjustable couch rails that are in the central location for the post‐oblique radiation fields can be seen through the Mylar foil of the tabletop.

Figure 1 shows the technique that was developed. The patient was positioned on a customized thin window Mylar top (thickness 0.3 mm) inserted in a Varian treatment couch. The couch top can be seen in Fig. 2. Six beam directions were chosen with gantry angles 60° apart as indicated in Fig. 1(b). In order to maximize source to skin distance (SSD) and therefore field size, the couch was moved in the direction opposite of the radiation beam. The maximal lateral motion of the couch was 20.8 cm which limited the displacement possible. In order to facilitate similar distances to patient central axis, couch height and lateral displacement were calculated using trigonometry taking the patient ant/post separation of 10 cm into consideration. Table 1 lists couch positions used relative to isocentre with the tan(30) × 20 cm = 11.54 rounded up to 12 cm.

**Table 1:** Initial couch positions relative to isocentre (0/0/0) for the six beam directions used in the work. The beam numbers are identical to the ones shown in Fig. 1(b). Positions were adjusted based on *in vivo* dosimetry as indicated in Table 2. In the initial setup, the distance to the patient midline is identical (124 cm) for each beam

Beam	Gantry		Couch position (cm, seen from feet)		
	Direction	Angle	Vertical	Longitudinal	Lateral
1	Ant	0	29 down	−30 or +30	0
2	Lt Ant Obl	60	17 down	−30 or +30	20 left
3	Lt Post Obl	120	7 up	−30 or +30	20 left
4	Post	180	19 up	−30 or +30	0
5	Rt Post Obl	240	7 up	−30 or +30	20 right
6	Rt Ant Obl	300	17 down	−30 or +30	20 right

After delivery of the first six fields to the upper part of the body, the patient was rotated and the lower half treated. Beam central axis location was verified by light field and the junction was marked on the patient's skin daily. The effective distance from the isocentre to the junction between the two sets of six beams was 30 cm, which was approximately at 65% dose in the electron beam profile of both beams assessed at 114 cm SSD and 1 mm depth under 30° oblique incidence reflecting “average” dose delivery conditions. The location of the junction was also informed by profiles acquired at perpendicular incidence and consideration of depth dose at different locations. Based on the *in vivo* dosimetry results, the location of the junction was later changed to 32 cm.

Electrons of 6 MeV nominal energy were employed using 36 × 36 cm^2^ jaw defined field size. No electron applicator was used but a 1.2 cm thick Poly(methyl‐methacrylate) (PMMA) block (density 1.16 g/cm^3^) mounted on the gantry to reduce the effective electron energy to approximately 3 MeV. High dose rate total skin electron mode (HDTSE 10 Gy per min at 1.6 m distance) setting was used to minimize the treatment time. The calibration of the beam is performed at 160 cm distance, which resulted in approximately 60 monitor units (MUs) to be given for each beam to ensure an incident dose of 1.5 Gy once all 12 beams are taken into account.

### 2.C Commissioning and verification measurements

Once the technique had been agreed upon, measurements were performed using several measurement setups and dosimetric equipment:

1. Output and depth dose were assessed using a PPC05 plane parallel chamber (IBA dosimetry, Schwarzenbrueck) and a thin window advanced Markus chamber (Exradin A10, Standard Imaging, Middleton, WI) in a solid water slab phantom.
2. A cylindrical PMMA phantom with density 1.16 g/cm^3^, length 20 cm and diameter 16 cm (shown in Fig. 2) was used for dose assessment around the circumference of the patient. This phantom was readily available as it is also used for assessment of Computed Tomography Dose Index in radiological procedures. Thermoluminescence dosimetry (TLD) using LiF:Mg,Cu,P was employed for the measurements.^15^, ^16^ TLD chips were individually calibrated and read out using an automatic TLD reader (Harshaw 5500, Thermo Fisher Scientific, Waltham, MA, USA). Standards were exposed using the same nominal energy as the patient irradiation (6 MeV electrons, exposed at depth of maximum dose). Other aspects of the measurement method are described in more detail by Lonski et al.^17^, ^18^
3. The head of an anthropomorphic phantom (ATOM dosimetry verification phantom, Computerized Imaging Reference Systems, CIRS, Norfolk Virginia) was used for radiochromic film measurements to assess dose in contour areas. The adult head phantom has similar dimensions to the body of a 2‐yr‐old child (20 × 15 cm^2^ cross section). Radiochromic film (EBT3, Ashland, Bridgewater, NJ, USA) was cut to fit into the phantom and exposed using approximately three times the dose to be delivered per fraction (150 MU per beam) to the patient to ensure relatively low doses could be assessed. Calibration films were exposed using the same nominal energy (6 MeV electrons at depth of maximum dose). The film was read using a flat bed scanner (EPSON 700) with 72 dpi resolution. The red channel with 16 bit depth was used for dosimetry.^19^

### 2.D *In vivo* dosimetry

**Figure 3:**
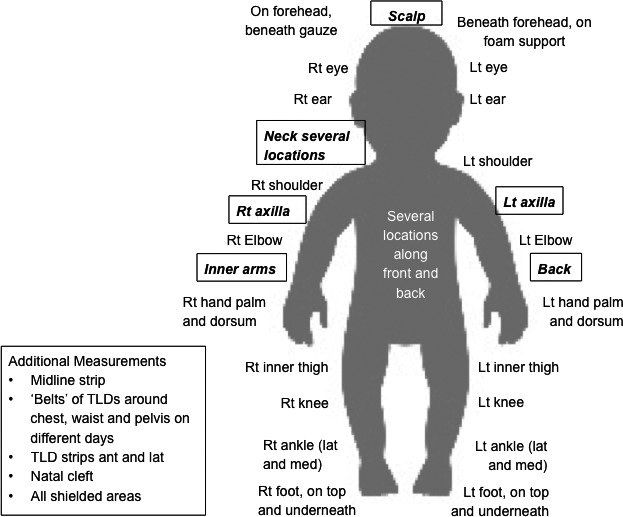
*In vivo* dosimetry locations for TLD. Most measurements were done only for selected fractions. The locations highlighted in *bold italics* were selected for boost treatments using conventional electron irradiation with appropriate bolus.

*In vivo* dosimetry was performed for most fractions using TLD and radiochromic film measurements using the same methods as described above. Figure 3 illustrates the measurement locations used. In addition to the point dose measurements indicated in the figure, rows of TLDs and strips of radiochromic film were used to identify dose across the junction of the two sets of fields and in locations where the edge of a boost field needed to be identified.

## 3 RESULTS

### 3.A Commissioning and verification measurements

Dose delivery was normalized to an ideal cylinder with 16 cm diameter (average diameter of the trunk of the patient) placed with the centre at 124 cm distance from the source for all fields delivered. Based on ion chamber measurements, 50 MU (HDTSE mode) was chosen per field resulting in a skin dose of approximately 1.44 Gy per fraction on a flat surface from all six beam directions by adding dose from fields with their respective incidence angle (eg, adding dose from beam perpendicular to the surface and twice the dose from a 60° angle).

**Figure 4:**
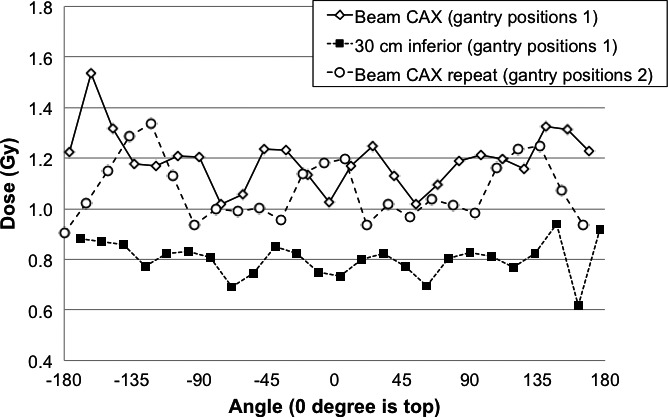
Results for the TLD ring around the PMMA phantom shown in Fig. 2. The results are shown for measurements for a single set of six fields delivered with 50 MU each with the ring and phantom at central axis and 30 cm away from central axis. Two beam arrangements are shown for a repeat measurement at central axis (gantry positions 1: 30°, 90°, 150°, 210°, 270° and 330°; gantry positions 2: 0°, 60°, 120°, 180°, 240° and 300° as illustrated in Fig. 1(b) and used in patient treatment).

Figure 4 shows the dose distribution in two locations around the PMMA phantom depicted in Fig. 2. The dose was assessed using TLDs 4 cm apart from each other on a strip around the phantom. Six beams of 50 MU each were delivered using the gantry angles depicted in Fig. 1(b). The TLD results effectively show dose at 1 mm depth^20^ and the uncertainty of each measurement is of the order of ±2% (1SD). The figure illustrates small variations with angle. Based on these measurements, the number of MUs was increased by 10% for the first fraction of the patient's treatment (HDTSE mode).

Dose was also assessed 30 cm from beam central axis (CAX) by moving the phantom 30 cm inferiorly. The average dose assessed confirms that the dose of each set of beams at the junction was 65% of the dose on CAX.

**Figure 5:**
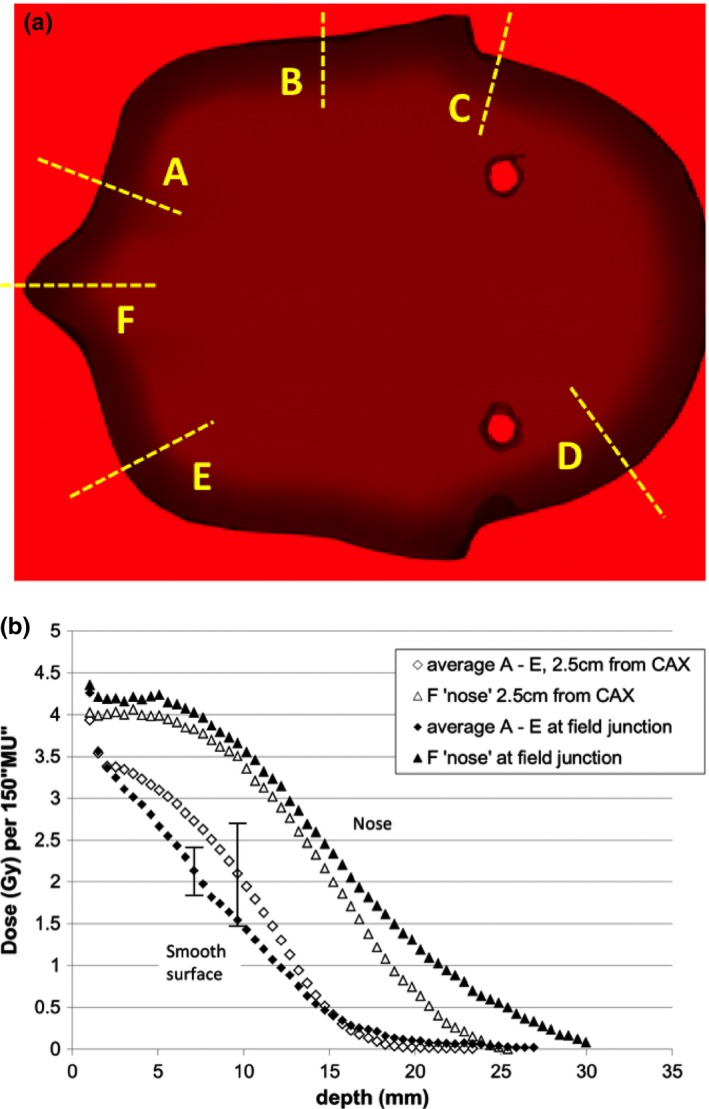
Radiochromic film measurement to determine depth dose in an anthropomorphic phantom. The head phantom was used to represent typical contours of a person with typical dimensions of a child. (a) Scan of a radiochromic film sheet cut to fit within the head phantom. Shown is the red color part of the spectrum, which was used for dosimetric evaluation. Also shown are six profile directions that were evaluated. (b) Depth dose profiles as per the directions shown in Fig. 5(a). The dose was scaled up by a factor of 3 (150 MU instead of 50 as per TLD measurements shown in Fig. 4) to allow for easier detection with EBT3 film. The results for scan directions A‐E are averaged in the figure with a typical variation shown for the two measurement positions 2.5 cm and 30 cm from CAX of the electron beams. The junction measurements were acquired using two sets of six electron beams.

The results of the film measurements in the anthropomorphic head phantom are shown in Fig. 5. Figure 5(a) pictures the red channel image of a transmission scan of EBT3 radiochromic film cut to fit within the head phantom. Also shown are six profile directions that were evaluated in the image. Depth dose measurements based on the red channel data along these profiles are shown in Fig. 5(b). The dose was scaled up approximately by a factor of 3 to allow for better signal‐to‐noise ratio. The results for scan directions A‐E are averaged in the figure with a typical variation shown for the two measurement positions 2.5 and 30 cm from CAX of the electron beams. The junction measurements were acquired using two sets of six electron beams.

It can be seen that due to the beam spoiler and the oblique incidence, the maximum dose is at the surface of the phantom with pointy parts of the anatomy receiving higher doses with deeper penetration.

Overall, the technique was found to provide ±20% dose homogeneity in a cylindrical phantom of 16 cm diameter and 20 cm length shifted longitudinally by 30 cm up with 50 MU per field (×12) in HDTSE delivering around 1.5 Gy incident dose. The patient was treated with 55 MU for each field in the first fraction taking ion chamber measurements and depth dose into account. Depth dose varies with location due to oblique incidence and curvature and is approximately 80% of the maximum dose at 5 mm depth and less than 10% beyond 15 mm.

### 3.B Patient treatment

Parental consent was obtained for treatment and all associated procedures as per our institutional practice. The patient commenced treatment when she was 31 months old. All 12 fractions of the treatment were delivered as planned. The patient was positioned as indicated in Section 2. A thin walled forced air warming blanket was used (“Bair hugger” 3M corporation) to reduce the risk of hypothermia for the patient. The dosimetric effect was assessed using a reduced set of TLDs on the cylindrical PMMA phantom and found to be negligible. Rotation of the patient between the two sets of six fields was done by manually rotating the patient on the thin window tabletop.

Typical duration for the treatment varied from 1.5 to 2 h with the patient under anesthetics for approximately 1‐1.5 h. The relatively long duration of treatment was due to the induction of anesthesia, the setup of the patient, the placement of TLDs, the need to move the couch between each field and the rotation of the patient on the Mylar support.

The treatment was tolerated well with only minor skin hypersensitivity and mild dry desquamation around the tangential areas of her neck and legs. At the 2‐week check, her hair was beginning to regrow. The patient relapsed systemically 2 months later and died 2 months after that. She never recurred in the skin.

### 3.C *In vivo* dosimetry

For nearly all fractions *in vivo* dosimetry was performed. Both TLD and radiochromic film were evaluated overnight prior to the next treatment fraction by relying on standards irradiated very close to the actual treatment time.

Thermoluminescence dosimetry *in vivo* dosimetry measurements over the first 6 days of treatment were used to improve the dose distribution and adjustments made to couch vertical height and number of monitor units per field as can be seen in Table 2. Dose to the back of the patient was low with lowering the couch resulting in somewhat improved dose.

**Table 2:** TLD measurement results for four locations on the patient's mid body. Shown are doses in Gy per fraction and the changes in treatment made based on the measurements. To account for the changes made, dose for 6 and 12 fractions was estimated by assuming each fraction without a measurement would yield the same dose as the previous one (indicated in parenthesis)

Fraction	Measured dose				Changes in technique based on *in vivo* dosimetry
	Ant	Left Lat	Right Lat	Post	
1	1.65	1.51	1.70	0.89	
2	(1.65)	(1.51)	(1.70)	(0.89)	Increase dose per field to 60 MU; increase separation at junction by 4 cm total
3	1.68	1.28	1.45	0.90	Couch lowered by 2 cm
4	(1.68)	1.59	1.53	1.04	Increase dose in anterior and posterior beams to 70 MU
5	1.67	(1.59)	(1.53)	0.79	Use 5 mm strip of 10 cm wide bolus on posterior side
6	1.89	1.53	1.64	1.12	No more bolus used; lower couch by an additional 2 cm
Total for 6 fractions	10.23	9.01	9.55	5.63	
Estimate for 12 fractions	21.6	18.2	19.4	12.4	

Eyes were shielded and partial shielding with PMMA was added to hands. All lead shielding was wrapped in thin plastic foil (“cling wrap”) to avoid direct contact with the skin. Also based on *in vivo* dosimetry, toenail shielding was used from the third fraction onwards while finger nail shielding was added after fraction 8. All shielding was manufactured in house and made from lead.

In total, 34 measurement points were taken in areas that were not shielded or considered for boost. The average dose of these points was 1.45 ± 0.29 Gy (20%, 1SD).

Based on patient geometry and the *in vivo* dosimetry measurements, six areas were identified for boost treatment using conventional electron irradiation fields:

- Scalp — 6 Gy in 3fx, 5 × 7 cm oval, 1.5 cm bolus, 110 cm SSD
- Lt Neck — 6 Gy in 3fx, 3.4 × 9 cm oval, 1.2 cm bolus, 110 cm SSD
- Rt Neck — 6 Gy in 3fx, 3.4 × 8 cm oval, 1.2 cm bolus, 110 cm SSD
- Lt Axilla — 6 Gy in 3fx, 3.4 × 10 cm oval, 1.2 cm bolus, 110 cm SSD
- Rt Axilla — 6 Gy in 3fx, 3.5 × 9 cm oval, 1.2 cm bolus, 110 cm SSD
- Posterior Back Strip — 4 Gy in 2fx, 30 × 8 cm rectangle, 1.5 cm bolus, 115 cm SSD

Additional areas assessed and not boosted were the perineum, the soles of feet, groins, natal cleft, the medial forearms and the chin fold.

## 4 DISCUSSION

The technique used here for pediatric TSET uses a similar approach to the Stanford technique for adults being based on two sets of six large field electron beams.^7^ However, using six gantry positions 60° apart, it was possible to treat a pediatric patient in supine position. By extending the SSD using a different couch position for each beam and employing two sets of fields 60 cm apart, it is possible to treat patients up to a body height of approximately 1 m. The field size chosen (36 × 36 cm^2^) was based on our technique for adults and could be modified.

*In vivo* dosimetry was found to be an essential part of the treatment approach monitoring progress and identifying opportunities to improve the dose distribution, which would have been difficult to predict with a cylindrical phantom. This has been reported by several authors for adult TSET^8^, ^21^, ^22^, ^23^ and in the present study more than 400 TLD chips have been read to ensure adequate dosimetry. The clinical situation did not allow for extensive commissioning exploring variations in patient size and shape. As such, some adjustment of the technique based on *in vivo* dosimetry was expected as Table 2 shows. In these modifications, we adopted a stepwise process to allow assessment of the dosimetric impact of changes made over the next fractions. Future treatments would benefit from this.

Not assessable by *in vivo* dosimetry are photon contamination and dose to internal critical structures such as bone. Photon contamination in the 6 MeV electron beam was assessed to be <0.5% of central axis maximum dose during commissioning. In a six field arrangement, this would limit the maximum photon dose in the centre of the patient to less than 3% of the incident dose. As many structures are closer to the skin in children than in adults, care was taken to optimize dose fall off as can be seen in Fig. 5(b). However, some dose to bone would have been inevitable but in general significantly lower than the skin dose. Shielding of hands reduced the dose in particularly thin areas where dose from both sides of the patients could contribute to internal structures.

The average dose measured in areas that were neither shielded nor considered for boost was 3% below the prescription dose (1.45 vs 1.5 Gy). However, even with attempts to improve the dose distribution based on *in vivo* dosimetry, homogenous dose distribution was difficult to achieve and dose variations exceeding 20% of the given dose can be expected. In particular, the dose to the back of the patients was lower than expected as shown in Table 1. As can be seen in Fig. 4, the low dose was not expected from a cylindrical phantom measurement. The position of the couch rails was adjusted to minimize their impact on dose, however, a shadowing effect may remain which would be different in patient and phantom due to different shape and Mylar support sag. The dose achieved by lowering the couch was deemed clinically acceptable.

On the other hand, the results presented here are likely to overestimate the dose inhomogeneity as the measurement locations were chosen to identify areas that were suspected to be high (eg, shoulders) or low (eg, inner thighs) doses. Compared to adult TSET, the variations were expected to be larger as the SSD is shorter thereby increasing the impact of surface contour variations. In addition to this, the need for a patient support structure introduces additional scatter and shadowing, which will be reflected in additional dose variation.

‘Although extensive commissioning is required, this treatment technique using a conventional linear accelerator can be delivered in a time slot of 1.75‐2 h for each fraction including time for setup, anesthetics, couch movements, and *in vivo* dosimetry.

## 5 CONCLUSION

Total skin electron therapy can be delivered to pediatric patients in supine position under general anesthetics using a conventional linear accelerator with a customized patient support. The treatment technique described here allows treatment of the whole skin of a young patient with acceptable dose accuracy but limited dose homogeneity. A considerable amount of work is required to commission the technique and *in vivo* dosimetry can inform personalization as required.

## CONFLICT OF INTEREST

None of the authors have any conflicts of interest to declare.
